# Tropical Diseases Research: Thirty Years and Counting

**DOI:** 10.1371/journal.pntd.0000329

**Published:** 2008-11-25

**Authors:** Peter J. Hotez

**Affiliations:** Sabin Vaccine Institute and Department of Microbiology, Immunology, and Tropical Medicine, George Washington University Medical Center, Washington, D.C., United States of America

TDR, the Special Programme for Research and Training in Tropical Diseases, was launched in 1978, more or less the same year I began my career in science as a Yale undergraduate working on the then nascent molecular biology of antigenic variation in African trypanosomes. Over the next two decades as a MD PhD student working on hookworms at Rockefeller University, and then back at Yale as a postdoctoral fellow and a member of the junior faculty there, I was told on multiple occasions that the likelihood of my making a career in scientific research on neglected tropical diseases was not very promising. After all, neglected diseases were neglected for a reason, including the fact that the most promising options for my obtaining long-term support at that time were (by today's standards) relatively modest funds from the Rockefeller Foundation, the John D. and Catherine T. MacArthur Foundation, the Burroughs Wellcome Fund (BWF), and the US National Institutes of Health (NIH) tropical medicine and parasitology study section. However, it turned out that through the establishment of some innovative networks, the stewards of those funding organizations were remarkably adept at leveraging those modest dollars into keeping alive a sustained effort for neglected tropical disease research. This carried the US neglected disease research community all the way until 1999, when funding scaled up dramatically with the entry of the Bill & Melinda Gates Foundation. Today, the support of the Gates Foundation is now being used to successfully leverage much of that earlier Rockefeller, BWF, MacArthur, Wellcome Trust, and NIH driven fundamental research into the development of new products and clinical testing for the major neglected tropical diseases.

Internationally, over the last 30 years the world has looked to TDR as a dependable supporter and innovator for research in tropical diseases. Through this period, the successes of almost all of my major overseas scientific collaborators depended heavily on TDR support, and TDR made it possible for literally hundreds of middle- and low-income country investigators to maintain meaningful scientific careers without leaving laboratories located in their native countries. TDR-funded research also provided a significant base for establishing our current generation of tropical disease products. Because of TDR and its joint activities with Rockefeller, Wellcome, and NIH, we now have in place an impressive overseas network of tropical disease research laboratories in Africa, Asia, and the Americas. The 250 peer-reviewed scientific papers published annually by TDR-supported scientists and laboratories (many of which appear regularly in *PLoS Neglected Tropical Diseases*) are a testament to good decision-making by the TDR and the productivity of its grantees. Today, these same TDR-supported laboratories are now playing a critical role in the product development and clinical testing activities supported by the Gates Foundation and other organizations. For example, our Human Hookworm Vaccine Initiative was able to hit the ground running upon receiving Gates Foundation funds in part because of strong overseas infrastructures established with the help of TDR support at the Rene Rachou Research Centre of the Oswaldo Cruz Foundation (FIOCRUZ) in Belo Horizonte, Brazil, and the Institute of Parasitic Diseases in Shanghai, China.

When it was launched 30 years ago, TDR had to be by necessity “everything to everybody,” meaning that they were one of the only games in town and laboratories everywhere depended on their support for basic science, translational research, product development, and clinical testing. However, as global health research support has increased and the 10/90 gap is slowly being bridged, this soup to nuts approach to tropical diseases is no longer necessary. This month in *PLoS Neglected Tropical Diseases*, we examine the shifting priorities of TDR and its future through four articles, including a summary of its Fourth External Review completed in 2006 by Professor Abdallah Daar and his colleagues [1], a viewpoint from the TDR leadership [2], including its director, Dr. Robert Ridley, and two commentaries written by Dr. Anthony Mbewu of South Africa's Medical Research Council and Drs. Daniel Carucci and Michael Gottlieb of the Foundation for the NIH, respectively [3],[4]. In an upcoming issue, we are publishing a Viewpoint from scientists in Kenya [5] (which complements an earlier August 2008 Viewpoint article by Drs. Garcia and Curioso from Peru [6]) on the challenges of retaining high quality scientists and maintaining scientific infrastructure in developing countries.

From these articles I can see some clear trends emerging. No organization, even well-resourced ones like the Gates Foundation, can any longer afford the luxury of being everything to everybody. The new global health initiatives and their mandate to fulfill Millennium Development Goal 6, “to combat HIV/AIDS, malaria and other diseases,” have simply become too big and too complex for any single organization to take on this entire task. In the interval year period between the Third and Fourth External Reviews, it could be argued that there was an unprecedented revolution in global health support and advocacy, one that brought in all of the government support of the G8 nations, massive private donor support, and a new generation of eager global health celebrities. It stands to reason, therefore, that the two reviews conducted in 1998 and 2006, respectively, might express opposite views. The first few years of the 21st century were a time of great transition in our field, and as the head of a product development partnership, I can certainly empathize with any leader who ran a global health organization during that time. It must be a particular challenge for the head of a United Nations organization like TDR, which by their complicated charter must appeal to a wide diversity of constituents.

In the article written by the TDR leadership, Ridley et al. express the view that because their organization works through partners in ways that promote their achievement, the achievements of TDR may sometimes be undervalued [2]. Those of us who have worked in the field of tropical medicine these last three decades deeply value the accomplishments of TDR. I also agree with their statement that TDR should be seen as a highly significant and successful entity, particularly “when judged against its budget.” At *PLoS Neglected Tropical Diseases*, we look forward to the coming years in order to see how the TDR responds to new challenges and mandates and retains their essential role in what has become a far more complicated and involved, albeit more interesting, space.
